# Identification and Synthesis of (*Z,Z*)-8,11-Heptadecadienyl Formate and (*Z*)-8-Heptadecenyl Formate: Unsaturated Aliphatic Formates Found in the Unidentified Astigmatid Mite, *Sancassania* sp. Sasagawa (Acari: Acaridae)

**DOI:** 10.3390/molecules21050619

**Published:** 2016-05-11

**Authors:** Nobuhiro Shimizu, Daisuke Sakata, Honami Miyazaki, Yasuhiro Shimura, Yasumasa Kuwahara

**Affiliations:** 1 Faculty of Bioenvironmental Science, Kyoto Gakuen University, 1-1 Nanjo, Sogabe, Kameoka 621-8555, Japan; d.sakata811@gmail.com (D.S.); kygf071220@yahoo.co.jp (H.M.); 2 Institute of Applied Biochemistry, The University of Tsukuba, 1-1-1 Tennodai, Tsukuba, Ibaraki 305-8577, Japan; shimizu1973@hotmail.co.jp; 3 Biotechnology Research Center and Department of Biotechnology, Toyama Prefectural University, 5180 Kurokawa, Imizu, Toyama 939-0398, Japan; kuwahara@pu-toyama.ac.jp; 4 Asano Active Enzyme Molecule Project, JST, ERATO, Imizu, Toyama 939-0398, Japan

**Keywords:** *Sancassania* sp. Sasagawa, mite, aliphatic formate, (*Z*,*Z*)-8,11-heptadecadienyl formate, (*Z*)-8-heptadecenyl formate, Barton decarboxylation

## Abstract

We identified two aliphatic formates, (*Z*,*Z*)-8,11-heptadecadienyl formate and (*Z*)-8-heptadecenyl formate in the opisthonotal gland secretions of an unidentified acarid species, namely *Sancassania* sp. Sasagawa. Both compounds were isolated using silica gel column chromatography and the structures were elucidated by ^1^H-NMR and GC/FT-IR. Further information on the double bond positions was obtained by GC-MS analysis of the corresponding dimethyl disulfide derivatives. Based on the estimated structures of the two formates and using linoleic and oleic acids as the respective starting materials, a simple four-step synthesis was achieved via Barton decarboxylation as the key step. The aliphatic formates identified in acarids thus far are neryl formate ((*Z*)-3,7-dimethylocta-2,6-dienyl formate) and lardolure (1,3,5,7-tetramethyldecyl formate), and both have been reported to have pheromone functions. The biological function of the two formates isolated in this study is currently being investigated. Although we can speculate that the two compounds were biosynthesized from linoleic and oleic acid, there is a possibility that the synthetic processes featured a novel chain shortening and formic acid esterification mechanism.

## 1. Introduction

All hydrocarbons observed in the opisthonotal gland secretions of acarid mites are straight-chain aliphatic compounds, and the chains are characteristically shorter than those of the hydrocarbons found in insects [1]. Carbon chains between C_13_ and C_17_ are particularly prominent, and while some of these compounds are known as pheromones, many are secreted as protective materials to act as solvents that control the evaporation of volatile compounds [2]. Hydrocarbons form through the elimination of the carboxyl group from fatty acids. (*Z,Z*)-6,9-Heptadecadiene, derived from linoleic acid (LA), and (*Z*)-8-heptadecene, derived from oleic acid (OA) are detected in a high proportion of acarid mites, yet there are almost no reports of their detection in insects [1]. As a result of isotope-labeled compound uptake experiments using *Carpoglyphus* *lactis*, the biosynthetic conversion of LA to (*Z,Z*)-6,9-heptadecadiene has been demonstrated [3].

Neryl formate ((*Z*)-3,7-dimethylocta-2,6-dienyl formate) and lardolure are aliphatic chain formates found in acarids. Neryl formate was the first alarm pheromone discovered in acarids [4] and was later found to function as an alarm pheromone in many types of mites [1]. Lardolure (1,3,5,7-tetramethyldecyl formate) has been identified as an aggregation pheromone in *Lardoglyphus* *konoi* and has been understood to present aggregation activation in other acarid mites such as *C*. *lactis*, *Aleuroglyphus* *ovatus*, and *Tyrophagus* *putrescentiae* [5,6,7]. Lardolure is a specific compound having a regular methyl side chain and is formed through the step-wise coupling of propionyl CoA with four molecules of methylmalonyl CoA [6]. The mechanism by which the carboxyl group is eliminated after the binding of the methylmalonyl CoA molecules and the subsequent formation of the formate remains unknown.

Two aliphatic formate compounds have now been detected in the secretions of the unidentified *Sancassania* sp. Sasagawa. While both compounds are presumed to be biosynthesized from the straight-chain unsaturated fatty acids LA and OA, as with lardolure, the carbon-chain shortening mechanism and formation of the formate ester require confirmation. This study reports the structure determination and efficient synthetic methodologies for preparing these two compounds.

## 2. Results and Discussion

**Figure 1:**
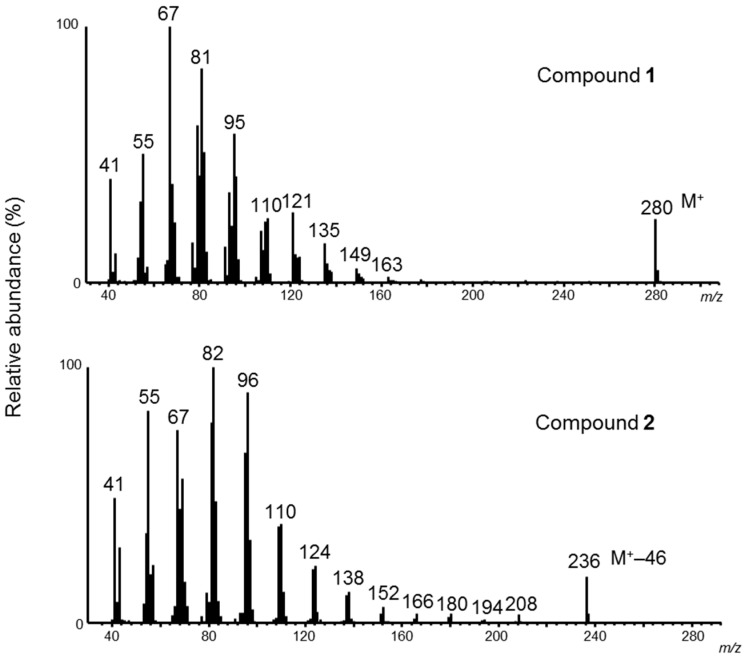
Mass spectra of compounds **1** and **2** in the secretions of *Sancassania* sp. Sasagawa.

When analyzing the hexane-extracted components of the opisthonotal gland secretions from *Sancassania* sp. Sasagawa by GC-MS, compounds **1** and **2** were detected at retention times of 18.912 min and 18.953 min, respectively. As presented in Figure 1, observation of the characteristic ion at *m*/*z* 280 and base peak ion at *m*/*z* 67 in the MS spectrum of compound **1** is possible, and from the *S*-shaped decrease in each fragment ion from the base ion to the *m*/*z* 280 ion, we speculated that compound **1** is a straight-chain fatty acid derived compound. The MS spectrum of compound **2** also shows an *S*-shaped decline in each fragment ion from the *m*/*z* 82 base peak ion to the characteristic *m*/*z* 236 ion, which led us to speculate that like compound **1**, compound **2** is a straight-chain fatty acid derived compound.

**Figure 2:**
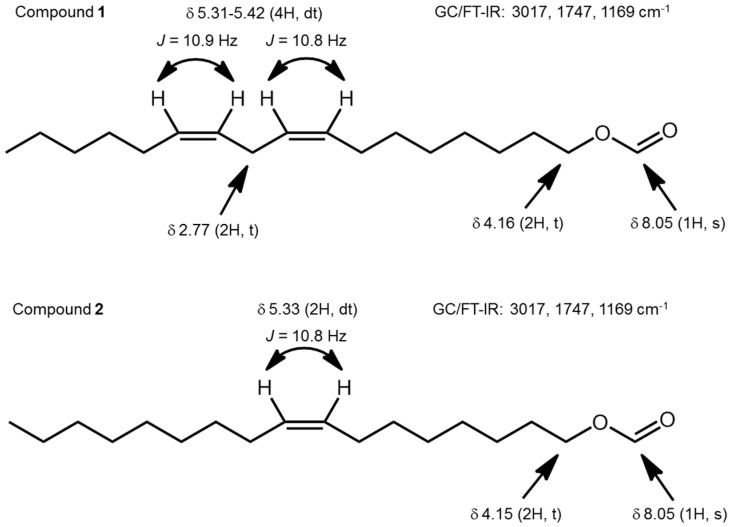
^1^H-NMR and GC/FT-IR analyses of natural compounds **1** and **2**.

To determine the structures of compounds **1** and **2**, the hexane extracts from the mites were purified on a silica gel column by step-wise changes of the composition of the elution solvent. The ^1^H NMR spectra of the isolated compounds **1** and **2** were then recorded (Figure 2). The signal at δ 8.05 (1H, s) for compound **1** was assigned to the aldehyde proton on the formate, while the signal at δ 5.31–5.42 (4H, dt, *J* = 10.8, 7.2 Hz, dt, *J* = 10.9, 7.1 Hz) was assigned to two Z-olefin protons, suggesting the existence of two double bonds in the molecule. Furthermore, the signal at δ 4.16 (2H, t) was assigned to the methylene protons adjacent to an oxygen atom, whereas the signal at δ 2.77 (2H, t, *J* = 6.2 Hz) was assigned to methylene protons sandwiched between two olefins.

In the ^1^H-NMR spectrum of compound **2**, the signals at δ 8.05 (1H, s) and δ 4.15 (2H, t) are similar to those seen in compound **1**, representing the aldehyde proton on the formate and methylene protons adjacent to an oxygen atom, respectively. However, the signal at δ 5.33 (2H, dt, *J* = 10.8, 7.0 Hz) suggests that there is only one Z-double bond in the molecule.

GC/FT-IR analysis of compounds **1** and **2** shows that they both exhibit an ester absorption band at 1747 cm^−1^ and an olefin absorption band at 3017 cm^−1^ (Figure 2). With regards to the *cis*-*trans* isomerism of compounds **1** and **2**, as the *E*-isomer specific absorption band near 980 cm^−1^ is not observed, the unsaturated bonds are assumed to be of the *Z*-form.

**Figure 3:**
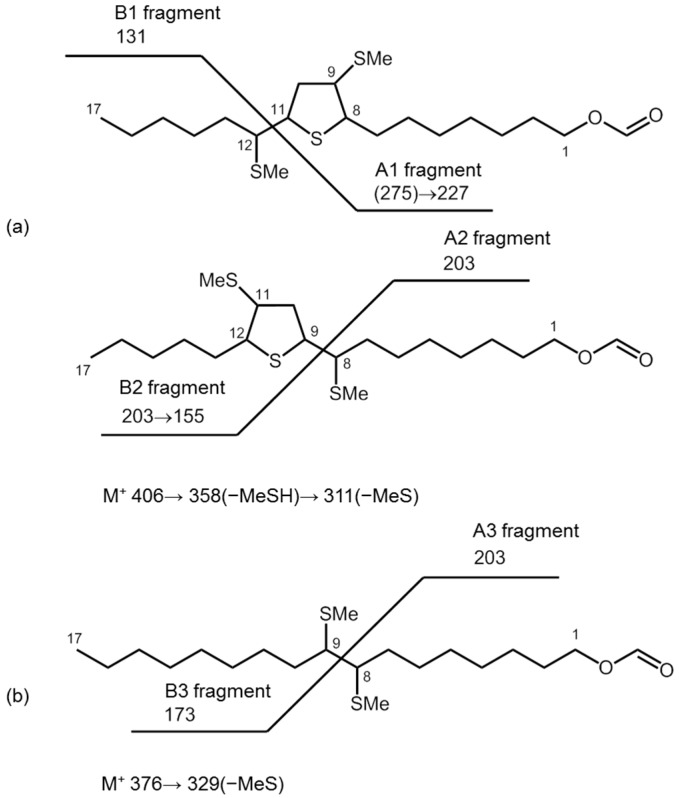
Mass fragmentation of DMDS derivatives derived from (**a**) compound **1** and (**b**) compound **2**.

To determine the double-bond positions within the molecules, compounds **1** and **2** were reacted with dimethyl disulfide (DMDS) and subjected to MS analysis [8]. If a diene compound contains one to three methylene groups between its double bonds, the addition of two molecules of DMDS to the double bonds affords a cyclic DMDS derivative through the elimination of dimethyl sulfide [2,9,10,11]. When subjecting the DMDS derivative of compound **1** to mass spectrometric analysis, as shown in Figure 3a, fragment ions are observed at *m*/*z* 227 (A1 fragment (C_13_H_23_O_2_S_2_)^+^-MeSH) and *m*/*z* 131 (B1 fragment (C_7_H_15_S)^+^), and the formation of tetrahydrothiophene (M^+^ *m*/*z* 406) from cyclization between positions 8 and 11, is detected. Furthermore, fragment ions are observed at *m*/*z* 203 (A2 fragment (C_10_H_19_O_2_S)^+^ or B2 fragment (C_10_H_19_S_2_)^+^) and *m*/*z* 155 (B2 fragment-MeSH), and we also identified tetrahydrothiophene (M^+^ *m*/*z* 406) from the cyclization between positions 9 and 12. We also observe a signal at *m*/*z* 358, which remains after the elimination of MeSH from the tetrahydrothiopyran (M^+^ *m*/*z* 406) cyclizing between positions 8 and 12, as well as at *m*/*z* 311, which remains after the elimination of MeS from the previous fragment ion. On the basis of these results, the double-bond position in compound **1** was determined to be between positions 8 and 11. When analyzing the DMDS derivative of compound **2**, the M^+^ ion at *m*/*z* 376, where one molecule of DMDS has added to the double bond, and the product resulting from elimination of MeS from this molecule, is observed at *m*/*z* 329. Furthermore, fragment ions at *m*/*z* 203 (A3 fragment (C_10_H_19_O_2_S)^+^) and *m*/*z* 173 (B3 fragment (C_10_H_21_S)^+^) are observed owing to the type of cleavage shown in Figure 3b. Thus, we concluded that there is a double bond at position 8. Based on all of the above spectral data, we conclude the structures of compounds **1** and **2** as (*Z*,*Z*)-8,11-heptadecadienyl formate and (*Z*)-8-heptadecenyl formate, respectively.

**Scheme 1:**
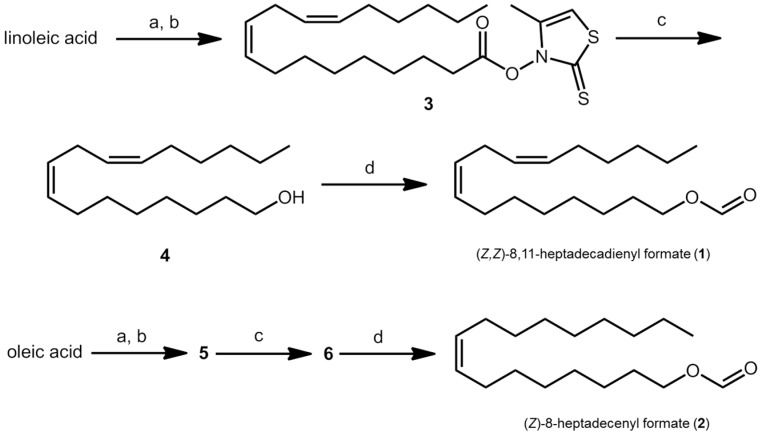
Synthesis of two formates (**1**) and (**2**). *Reagents and conditions*: (**a**) oxalyl chloride, DMF, benzene; (**b**) 3-hydroxy-4-methyl-2(3*H*)-thiazolethione, pyridine, Et_2_O, 74% (2 steps); (**c**) *tert*-dodecanethiol, O_2_, visible light, triphenylphosphine, 41%; (**d**) formic acid, acetic anhydride, pyridine, 69%.

Compounds **1** and **2** were each synthesized in a four-step process, as shown in Scheme 1, with Barton decarboxylation being the key reaction [12,13].

Oxalyl chloride was used to convert the starting material LA to its acyl chloride form, and ester **3** was formed by reacting the acyl chloride with 3-hydroxy-4-methyl-2(3*H*)-thiazolethione. Ester **3** was irradiated with light in the presence of *tert*-dodecanethiol under oxygen atmosphere and reductively worked-up to produce the alcohol **4**. By forming the formate from alcohol **4**, the target formate compound **1** is obtained in a total yield of 21%. 

In previously reported work, two rounds of Grignard coupling between a terminal alkyne and an alkyl halide were employed to extend the carbon chain, after which Lindlar reduction was used to obtain alcohol **4**. The whole procedure featured a total of six steps, and an overall yield of 18% [14]. By using the readily available and cheap unsaturated fatty acid LA as the starting material, the synthetic route developed in this study did not require carbon-carbon bond formation nor control of geometric isomerism, thereby achieving the facile synthesis of alcohol **4** in three steps and with an overall yield of 30%.

Formate **2** was easily prepared using OA as the starting material following the same synthetic route used for compound **1**. As the GC retention times, MS spectra, and ^1^H-NMR spectra of the synthesized compounds **1** and **2** exactly match those of the natural products, compound **1** and compound **2** are confirmed as (*Z*,*Z*)-8,11-heptadecadienyl formate and (*Z*)-8-heptadecenyl formate, respectively.

Although the formate compounds **1** and **2** have been reported as preorbital secretions in mammals such as the Cape grysbok (*Raphicerus* *melanotis*), male oribi (*Ourebia* *ourebi*) and male suni (*Neotragus* *moschatus*) [15,16,17], this study presents the first example of their identification in arthropods. Furthermore, while the above reports concerned only the structure determination of the compounds, our work enables the quantitative examination of the bioactivity of compounds **1** and **2** owing to the successful achievement of their synthesis.

It can be surmised from the double-bond positions that formate **1** is biosynthesized from LA, while compound **2** comes from OA. Isotope-labeled-compound uptake studies using *C*. *lactis* have demonstrated that (*Z*,*Z*)-6,9-heptadecadiene, a hydrocarbon that is also a secretion component of *Sancassania* sp. Sasagawa, is biosynthesized from LA [3]. It has been reported that the biosynthesis of hydrocarbons in insects works through the activity of cytochrome P450 on the aldehydes that result from the reduction of fatty acyl-CoA [18,19,20,21]. Assuming that the same pathway is followed in acarids, it is thought that (*Z,Z*)-6,9-heptadecadiene is synthesized with the aldehyde derived from LA (linoleoyl-CoA) being the precursor, and work is currently underway to identify the enzyme involved in this conversion. As the formate compounds **1** and **2** are secreted together with the hydrocarbons (*Z,Z*)-6,9-heptadecadiene and (*Z*)-8-heptadecene, they are thought to be synthesized through the activity of different enzymes on a common precursor within the secretory glands. However, the mechanism of this conversion remains unknown. To resolve this problem, we are currently performing detailed spectral analysis of formate compounds **1** and **2**, following isotope-labeled-compound uptake by *Sancassania* sp. Sasagawa.

## 3. Experimental

### 3.1. General Procedures

Column chromatography was performed on a Wakosil silica gel C-200 column with the specified solvents. ^1^H- and ^13^C-NMR spectra were recorded on either a Biospin AC400M spectrometer (400 MHz for ^1^H and 100 MHz for ^13^C, Bruker, Yokohama, Japan) or on a Bruker 500 MHz (500 MHz for ^1^H and 125 MHz for ^13^C) using tetramethylsilane as the internal standard. Natural compounds, DMDS derivatives, and synthetic compounds were analyzed using the following two gas liquid chromatograph systems; a model 263-30 (Hitachi, Tokyo, Japan) in split mode using a Al-clad fused capillary column (0.25 mm i.d. × 25 m, 0.1 μm film thickness, Quadrex, Bethany, CT, USA) using a temperature gradient from 135 to 250 °C at 4 °C/min, and a 6890N (Agilent Technologies Inc., Santa Clara, CA, USA) in splitless mode using an HP-5MS capillary column (0.25 mm i.d. × 30 m, 0.25 μm film thickness, Agilent Technologies Inc.) using a temperature gradient from 60 °C (2 min) to 290 °C (5 min hold) at 10 °C/min. Mass spectra were measured using either a Hitachi M-80B high-resolution mass spectrometer operated at 70 eV or an Agilent Technologies 5975 Inert XL mass selective detector operated at 70 eV. IR spectra were recorded using a FTIR 4300 (Shimadzu, Kyoto, Japan) with an OV-1 bonded wide-bore column (0.53 mm i.d. × 25 m, 5 μm film thickness, GL Sciences, Tokyo, Japan).

### 3.2. Mites

*Unidentified Sancassania* sp. Sasagawa (Acari: Acaridae) is a strain derived as the hypopus attached to the Japanese rhinoceros beetle *Trypoxylus dichotomus* (L. 1771). The culture lines were maintained for generations using the following rearing conditions: dry yeast feeding at 25 °C and 90% relative humidity and on agar medium as reported previously [22] at 20 °C.

### 3.3. Extraction and Isolation

Mites (85.9 g) were separated from the culture medium and extracted with hexane (300 mL) to obtain the secretions of the opisthonotal glands. After evaporation of the solvent, the extract (73 mg) was applied to a SiO_2_ column (500 mg, Wako-gel C-200) and successively eluted with 5.0 mL of hexane, and mixtures of ether in hexane (3%, 5%, 10%, and 20%). A mixture of formates **1** and **2** eluted with 3% ether in hexane, was applied to a SiO_2_ column (500 mg, Wako-gel C-200), and eluted with 1.0 mL of mixtures of benzene in hexane (25% and 50%). Both **1** and **2** eluted with 25% benzene in hexane were further purified by column chromatography using a SiO_2_ column (500 mg, Wako-gel C-200) and eluted with 10% benzene in hexane to yield **1** (4.2 mg) and **2** (1.1 mg) separately.

### 3.4. Determination of Double-Bond Positions in Formates ***1*** and ***2***

The positions of the double bonds in formates **1** and **2** were determined from the MS spectra of the DMDS derivatives, following a previously reported method [8]. Compound **1** was dissolved in DMDS (200 μL) with a catalytic amount of I_2_ and kept overnight at 60 °C. The DMDS product was extracted with hexane and worked up as reported elsewhere [2]. The DMDS derivative of compound **2** was prepared according to the same procedure as that used for compound **1**.

### 3.5. Synthesis

#### 3.5.1. 4-Methyl-2-thioxothiazol-3(2*H*)-yl(*Z*,*Z*)-9,12-octadecadienoate (**3**)

To a solution of LA (1.0 g, 3.57 mmol) in benzene (10 mL), oxalyl chloride (1.0 mL, 11.7 mmol) and DMF (1 drop) was added at room temperature. The mixture was stirred for 2 h and then evaporated *in vacuo*. The residue was dissolved in diethyl ether (3 mL) and the mixture was added to a solution of 3-hydroxy-4-methyl-2(3*H*)-thiazolethione (535 mg, 3.64 mmol) and pyridine (2 drops) in diethyl ether (12 mL). The mixture was stirred for 10 min at room temperature, after which it was filtered and evaporated *in vacuo*. The residue was purified on a SiO_2_ column (hexane:EtOAc, 6:1) to yield ester **3** as a bright yellow oil (1.08 g, 74%). ^1^H-NMR (400 MHz, CDCl_3_) δ 6.23 (1H, d, *J* = 1.2 Hz), 5.36 (4H, m), 2.76 (2H, t, *J* = 6.0 Hz), 2.71 (1H, t, *J* = 8.6 Hz), 2.64 (1H, t, *J* = 8.0 Hz), 2.16 (3H, d, *J* = 1.2 Hz), 2.06 (4H, m), 1.82 (2H, m), 1.46 (2H, m), 1.24–1.39 (12H, m), 0.89 (3H, t, *J* = 6.8 Hz). ^13^C-NMR (100 MHz, CDCl_3_) δ 180.85 (C=S), 168.96 (C=O), 136.90 (=C–N), 130.25 (C=), 129.96 (C=), 128.14 (C=), 127.89 (C=), 102.37 (=C–S), 31.53 (CH_2_), 31.36 (CH_2_), 29.56 (CH_2_), 29.35 (CH_2_), 29.02 (CH_2_), 28.97 (CH_2_ × 2), 27.21 (CH_2_), 27.17 (CH_2_), 25.65 (CH_2_), 24.59 (CH_2_), 22.58 (CH_2_), 14.08 (CH_3_), 13.35 (CH_3_).

#### 3.5.2. 4-Methyl-2-thioxothiazol-3(2*H*)-yl(*Z*)-9-octadecenoate (**5**)

Ester **5** was prepared from OA in the same manner as ester **3**. ^1^H-NMR (400 MHz, CDCl_3_) δ 6.24 (1H, d, *J* = 1.2 Hz), 5.35 (2H, m), 2.71 (2H, m), 2.16 (3H, d, *J* = 1.2 Hz), 2.02 (4H, m), 1.82 (2H, m), 1.24–1.45 (20H, m), 0.88 (3H, t, *J* = 6.8 Hz). ^13^C-NMR (100 MHz, CDCl_3_) δ 180.82 (C=S), 168.97 (C=O), 136.90 (=C–N), 130.09 (C=), 129.66 (C=), 102.39 (=C–S), 31.91 (CH_2_), 31.35 (CH_2_), 29.77 (CH_2_), 29.65 (CH_2_), 29.53 (CH_2_), 29.33 (CH_2_ × 2), 29.02 (CH_2_ × 2), 28.97 (CH_2_), 27.23 (CH_2_), 27.14 (CH_2_), 24.59 (CH_2_), 22.69 (CH_2_), 14.13 (CH_3_), 13.36 (CH_3_).

#### 3.5.3. (*Z*,*Z*)-8,11-Octadecadienol (**4**)

A solution of ester **3** (100 mg, 0.244 mmol) in toluene (12 mL) containing *tert*-dodecanethiol (198 mg, 0.980 mmol) was stirred vigorously and irradiated under oxygen atmosphere using an incandescent lamp (60 W). After stirring at room temperature overnight, triphenylphosphine (96 mg, 0.366 mmol) was added and the mixture was stirred for several minutes. Evaporation of the solvent yielded a residual oil that upon purification by SiO_2_ column chromatography (hexane:EtOAc, 4:1) afforded alcohol **4** (25 mg, 41%). ^1^H-NMR (400 MHz, CDCl_3_) δ 5.39 (4H, m), 3.64 (2H, t, *J* = 6.4 Hz), 2.77 (2H, t, *J* = 6.8 Hz), 2.05 (4H, m), 1.57 (2H, m), 1.24–1.39 (14H, m), 0.89 (3H, t, *J* = 6.8 Hz). ^13^C-NMR (100 MHz, CDCl_3_) δ 130.23 (C=), 130.07 (C=), 128.05 (C=), 127.92 (C=), 63.09 (CH_2_O), 32.79 (CH_2_), 31.54 (CH_2_), 29.60 (CH_2_), 29.35 (CH_2_), 29.32 (CH_2_), 29.25 (CH_2_), 27.21 (CH_2_ × 2), 25.71 (CH_2_), 25.64 (CH_2_), 22.58 (CH_2_), 14.07 (CH_3_). MS (EI) *m*/*z* (rel. %): 252 (M^+^, 4), 234 (1), 149 (5), 135 (11), 121 (13), 109 (24), 95 (59), 81 (84), 67 (100), 55 (50), 41 (40).

#### 3.5.4. (*Z*)-8-Octadecenol (**6**)

Alcohol **6** was prepared from ester **5** in the same manner as alcohol **4**. ^1^H-NMR (400 MHz, CDCl_3_) δ 5.35 (2H, m), 3.65 (2H, t, *J* = 6.8 Hz), 2.02 (4H, m), 1.57 (2H, quint, *J* = 7.2 Hz), 1.27 (20H, m), 0.88 (3H, t, *J* = 7.2 Hz). ^13^C-NMR (100 MHz, CDCl_3_) δ 130.02 (C=), 129.78 (C=), 63.10 (CH_2_O), 32.78 (CH_2_), 31.92 (CH_2_), 29.78 (CH_2_), 29.70 (CH_2_), 29.54 (CH_2_), 29.34 (CH_2_ × 2), 29.26 (CH_2_), 29.09 (CH_2_), 27.23 (CH_2_), 27.19 (CH_2_), 25.72 (CH_2_), 22.71 (CH_2_), 14.15 (CH_3_). MS (EI) *m*/*z* (rel. %): 254 (M^+^, <1), 236 (11), 208 (3), 152 (4), 137 (9), 123 (17), 109 (34), 96 (76), 82 (100), 67 (73), 55 (77), 41 (44).

#### 3.5.5. (*Z*,*Z*)-8,11-Heptadecadienyl Formate (**1**)

To alcohol **4** (25 mg, 0.099 mmol) in pyridine (0.5 mL), a mixed anhydride (210 μL), which was prepared by mixing acetic anhydride (60 μL) and formic acid (150 μL) at 0 °C, was added dropwise at 0 °C. The mixture was maintained at this temperature for 4 h. Iced water was then added to the solution, which was extracted with EtOAc. The organic layer was successively washed with 2*N* HCl, saturated NaHCO_3_, and brine and dried over Na_2_SO_4_. After evaporation of the solvent *in vacuo*, the resulting oil was purified by SiO_2_ column chromatography (hexane:EtOAc, 4:1) to give formate **1** (19 mg, 69%). ^1^H-NMR (400 MHz, CDCl_3_) δ 8.06 (1H, s), 5.37 (4H, dt, *J* = 10.8, 7.2 Hz, dt, *J* = 10.9, 7.1 Hz), 4.16 (2H, t, *J* = 7.2 Hz), 2.77 (2H, t, *J* = 6.2 Hz), 2.05 (4H, m), 1.65 (2H, m), 1.26–1.40 (14H, m), 0.89 (3H, t, *J* = 6.8 Hz). ^13^C-NMR (100 MHz, CDCl_3_) δ 161.21 (OCHO), 130.25 (C=), 129.99 (C=), 128.12 (C=), 127.89 (C=), 64.11 (CH_2_O), 31.54 (CH_2_), 29.54 (CH_2_), 29.35 (CH_2_), 29.13 (CH_2_), 29.09 (CH_2_), 28.50 (CH_2_), 27.21 (CH_2_), 27.17 (CH_2_), 25.80 (CH_2_), 25.64 (CH_2_), 22.58 (CH_2_), 14.07 (CH_3_). HRMS *m*/*z* (M^+^). Calculated for C_18_H_32_O_2_: 280.2400; Found 280.2379.

#### 3.5.6. (*Z*)-8-Heptadecenyl formate (**2**)

Formate **2** was prepared from alcohol **6** in the same manner as formate **1**. ^1^H-NMR (400 MHz, CDCl_3_) δ 8.06 (1H, s), 5.35 (2H, dt, *J* = 10.8, 7.0 Hz), 4.16 (2H, t, *J* = 6.8 Hz), 2.02 (4H, m), 1.66 (2H, quint, *J* = 6.8 Hz), 1.22–1.40 (20H, m), 0.88 (3H, t, *J* = 7.2 Hz). ^13^C-NMR (100 MHz, CDCl_3_) δ161.23 (OCHO), 130.06 (C=), 129.68 (C=), 64.11 (CH_2_O), 31.91 (CH_2_), 29.76 (CH_2_), 29.63 (CH_2_), 29.53 (CH_2_), 29.33 (CH_2_ × 2), 29.12 (CH_2_), 29.09 (CH_2_), 28.49 (CH_2_), 27.22 (CH_2_), 27.14 (CH_2_), 25.79 (CH_2_), 22.69 (CH_2_), 14.13 (CH_3_). HRMS *m*/*z* (M^+^–HCOOH). Calculated for C_17_H_32_: 236.2502; Found 236.2481.

## 4. Conclusions

We have achieved a facile synthesis of the two formates, (*Z*,*Z*)-8,11-heptadecadienyl formate and (*Z*)-8-heptadecenyl formate, which are the main secretions from the acarid *Sancassania* sp. Sasagawa, with the Barton decarboxylation being a key reaction in the synthesis. While the position of their double bonds suggests that their biosynthesis proceeds from linoleic or oleic acid, respectively, their biosynthetic pathways may feature a novel chain-shortening mechanism and formate formation.
